# Thrombose veineuse cérébrale révélant une maladie de Behcet chez un enfant

**DOI:** 10.11604/pamj.2015.20.259.6321

**Published:** 2015-03-17

**Authors:** Sahar Messaoudi, Maria Rkain, Anass Es Seddiki, Khalid Serraj, Rim Amrani, Noufissa Benajiba

**Affiliations:** 1 Service de Pédiatrie, CHU Mohamed VI, Oujda, Maroc; 2 Service de Médecine Interne, CHU Mohamed VI, Oujda, Maroc

**Keywords:** Behçet, thrombose veineuse cérébrale, IRM, Fond d′œil, enfant, Behçet, cerebral veinous thrombosis, MRI, Fundus, child

## Abstract

La maladie de Behçet est une vascularite systémique, évoluant par poussées. Son diagnostic est clinique: les manifestations les plus fréquentes sont l'aphtose buccale et génitale et l'uvéite. On peut également observer des atteintes neurologiques, digestives, vasculaires, articulaires ou cutanées. Nous rapportons une observation de maladie de Behçet révélée par une thrombose veineuse cérébrale.

## Introduction

La maladie de Behçet est une vascularite systémique, évoluant par poussées. Son diagnostic est clinique: les manifestations les plus fréquentes sont l'aphtose buccale et génitale et l'uvéite [1, 2]. On peut également observer des atteintes neurologiques, digestives, vasculaires, articulaires ou cutanées. Les cas associant maladie de Behçet et singnes neurologiques ont été rapportés chez des enfants qui présentaient des céphalées (associées à un œdème papillaire); la diminution de l'acuité visuelle a été rarement décrite [1, 2]. L'IRM cérébrale semble être le meilleur moyen pour faire le diagnostic de la thrombose veineuse cérébrale [1, 2]. Nous rapportons une observation de maladie de Behçet révélée par une thrombose veineuse cérébrale chez un enfant de dix ans.

## Patient et observation

Il s'agit d'un enfant de sexe masculin, âgé de dix ans se plaignant depuis six mois de céphalées partiellement soulagées par du paracétamol et quatre épisodes d'aphtose buccale et périnatale évoluant depuis une année. Il a été hospitalisé pour syndrome méningé apyrétique. L'examen clinique a retrouvé une raideur de nuque; le reste de l'examen, en particulier neurologique était sans particularité. La tomodensitométrie cérébrale sans injection était en faveur d'une thrombose veineuse cérébrale. L'analyse du liquide céphalorachidien (LCR) montrait une normocytose à moins de 3 leucocytes (GB)/µl avec une protéinorachie à 0,40 g/l et une glycorachie à 0,75 mmol/l avec une glycémie à 1,50 g/l concomitante. Il a été traité par anticoagulant par voie intraveineuse (IV). Après disparition des céphalées durant quelques jours, l'examen clinique était normal mais il existait un syndrome inflammatoire biologique avec vitesse de sédimentation (VS) à 65 mm. L'examen du fond d’œil (FO) a mis en évidence un œdème papillaire bilatéral; l'acuité visuelle était conservée.

Au cours de l'hospitalisation, l'enfant a présenté pour la cinquième fois des lésions aphtoïdes anales et buccales et on a noté l'apparition retardée de papulopustules aux sites de ponction veineuse; signe d'hypersensibilité aux points de piqûre, ce qui a amené à évoquer le diagnostic de maladie de Behçet. Une tomodensitométrie cérébrale (avec injection), mettait en évidence une thrombose du sinus longitudinal supérieur avec reperméabilisation partielle et une thrombose de plusieurs veines corticales. Un traitement par prednisone (2 mg/kg/j) et l'azathioprine (10 mg/semaine) a été institué, associé à un traitement par aspirine à doses anti-agrégantes; un traitement anticoagulant a été prescrit. Les céphalées ont régressé rapidement. Le bilan de thrombose (protéines C, S, et antithrombine III, anticoagulant circulant, test de résistance à la protéine C activée, mutation du gène du facteur II) était normal. Le syndrome inflammatoire a disparu après trois semaines de traitement. Le FO s'est normalisé en quatre mois.

## Discussion

Il n'existe pas de signe biologique spécifique de la maladie de Behçet dont le diagnostic est essentiellement clinique. L'International Study Group for Behçet's Disease (ISGBD) a proposé en 1990 des critères diagnostiques [1]: des ulcérations buccales récurrentes (au moins trois fois en 12 mois) doivent être présentes, ainsi qu'au moins deux des critères suivants: ulcérations génitales récurrentes; lésions oculaires: uvéite, hyalite ou vascularité rétinienne; lésions cutanées (érythème noueux, pseudofolliculite, lésions papulopustuleuses ou nodules acnéiformes); signe de pathergie (réaction d'hypersensibilité avec apparition d'une lésion papulopustuleuse 12 à 48 heures après une ponction sous-cutanée ou intraveineuse). Cette définition a fait l'objet de critiques, car elle reflète mal les formes de début chez l'enfant [2–4] et ne prend pas en compte certains aspects de la maladie de Behçet, notamment les thromboses veineuses et l'atteinte inflammatoire neuroméningée, retenues comme critères diagnostiques dans certaines définitions (critères d'O'Duffy et Goldstein, et critères de Mason et Barnes) [2]. Notre observation illustre bien ces critiques car, le diagnostic de maladie de Behçet ne pouvait pas être retenu sur la base des seuls critères de l'ISGBD.

Les manifestations neurologiques chroniques sont relativement fréquentes dans la maladie de Behçet. Chez l'adulte, elles ont été notées dans 10 à 20% des cas (43% si on intègre les céphalées isolées) [5]. Dans la série pédiatrique de Koné-Paut et Bernard, leur fréquence était de 27%, 42% avec les céphalées isolées [6]. L'atteinte neurologique peut correspondre à une méningite, à une méningo-encéphalite ou à une thrombose veineuse cérébrale [4] et les anciennes publications « d'hypertension intracrânienne bénigne » correspondaient probablement à des thromboses veineuses cérébrales non documentées. Plusieurs cas associant maladie de Behçet et hypertension intracrânienne, comme dans notre observation, ont été rapportés chez l'enfant [7–10]. Tous ces patients présentaient des céphalées, en général associées à un œdème papillaire Rarement, ils souffraient de diminution de l'acuité visuelle, ce qui est le cas pour notre patient. L'analyse du LCR montrait inconstamment une hypercytose. Actuellement, l'IRM cérébrale semble être le meilleur moyen pour faire le diagnostic de thrombose veineuse cérébrale, pour notre malade on n'a pas pu faire une IRM cérébrale par manque de moyen. En cas de thrombophlébite, la corticothérapie a un effet spectaculaire, avec régression rapide des céphalées, et de l’œdème papillaire en quelques mois, chez notre patient les céphalées ont régressé au bout d'une semaine alors que l’œdème papillaire n'a disparu que vers la fin de la deuxième semaine. Un traitement anticoagulant est indiqué en cas de thrombose récente, sans signes de reperméabilisation. Le pronostic neurologique des thromboses veineuses cérébrales est favorable, mais il existe un risque de récidive, justifiant un traitement préventif par anti-agrégant plaquettaire [8].

## Conclusion

Cette observation nous incite à penser à une maladie de Behcet devant l'association d'une aphtose buccale et génitale récidivante et une thrombose veineuse cérébrale. Les critères diagnostiques proposés par l'ISGBD permettent souvent de retenir le diagnostic de maladie de Behçet chez l'enfant. Mais il est alors important de prendre en compte l'atteinte neurologique (méningo-encéphalite ou thrombose veineuse cérébrale) comme critère diagnostique additionnel.

## References

[1] International Study Group for Behçet's Disease (1990). Criteria for diagnosis of Behçet's disease. Lancet.

[2] Petty RE, Cassidy JT (2001). Behçet's disease and other vasculitis. Textbook of Pediatric Rheumatology.

[3] Koné-Paut I (1999). Maladie de Behçet. Rhumatologie pédiatrique.

[4] Hamza M, Kahn MF, Peltier AP, Meyer O, Piette JC (2000). Maladie de Behçet. Maladies et syndromes systémiques.

[5] Filali Ansary N, Tazi Mezalek Z, Mohattane A, Adnaoui M, Aouini M, Maaouni A (1999). La maladie de Behçet: 162 observations. Ann Med Interne.

[6] Koné-Paut I, Bernard JL (1993). La maladie de Behçet chez l'enfant. Arch Fr Pédiatr..

[7] Ibrahimi A, Ouammou A, Assamti O, Mouine A, El Ouarzazi A (1983). Hypertension intracrânienne dite « bénigne » et maladie de Behçet. Neurochirurgie..

[8] Humberclaude V, Vallée L, Sukno S, Vamecq J, Dhellemmes S, Pruvo JP (1993). Thrombose veineuse cérébrale révélant une maladie de Behçet. Arch Fr Pédiatr..

[9] Pamir MN, Kansu T, Erbengi A, Zileli T (1981). Papilledema in Behçet'syndrome. Arch Neurol..

[10] Koné-Paut I, Yurdakul S, Bahabri A, Shafae N, Ozen S, Ozdogan H (1998). Clinical features of Behçet's disease in children: An international collaborative study of 86 cases. J Pediatr..

